# Estimating malaria burden among pregnant women using data from antenatal care centres in Tanzania: a population-based study

**DOI:** 10.1016/S2214-109X(19)30405-X

**Published:** 2019-12

**Authors:** Chonge Kitojo, Julie R Gutman, Frank Chacky, Emmanuel Kigadye, Sigsbert Mkude, Renata Mandike, Ally Mohamed, Erik J Reaves, Patrick Walker, Deus S Ishengoma

**Affiliations:** US President’s Malaria Initiative, USAID, Dar es Salaam, Tanzania; Open University of Tanzania, Dar es Salaam, Tanzania; Malaria Branch, Division of Parasitic Diseases and Malaria, Center for Global Health, Centers for Disease Control and Prevention, Atlanta, GA, USA; National Malaria Control Programme, Dodoma, Tanzania; Open University of Tanzania, Dar es Salaam, Tanzania; National Malaria Control Programme, Dodoma, Tanzania; National Malaria Control Programme, Dodoma, Tanzania; National Malaria Control Programme, Dodoma, Tanzania; Malaria Branch, Division of Parasitic Diseases and Malaria, Center for Global Health, Centers for Disease Control and Prevention, and US President’s Malaria Initiative, Dar es Salaam, Tanzania; MRC Centre for Global Infectious Disease Analysis, Department of Infectious Disease Epidemiology, Imperial College London, London, UK; National Institute for Medical Research, Tanga Research Centre, Tanga, Tanzania

## Abstract

**Background:**

More timely estimates of malaria prevalence are needed to inform optimal control strategies and measure progress. Since 2014, Tanzania has implemented nationwide malaria screening for all pregnant women within the antenatal care system. We aimed to compare malaria test results during antenatal care to two population-based prevalence surveys in Tanzanian children aged 6–59 months to examine their potential in measuring malaria trends and progress towards elimination.

**Methods:**

Malaria test results from pregnant women screened at their first antenatal care visits at health-care facilities (private and public) in all 184 districts of Tanzania between Jan 1, 2014, and Dec 31, 2017, were collected from the Health Management Information Systems and District Health Information System 2. We excluded facilities with no recorded antenatal care attendees during the time period. We standardised results to account for testing uptake and weighted them by the timing of two population-based surveys of childhood malaria prevalence done in 2015–16 (Demographic and Health Survey) and 2017 (Malaria Indicator Survey). We assessed regional-level correlation using Spearman’s coefficient and assessed the consistency of monthly district-level prevalence ranking using Kendall’s correlation coefficient.

**Findings:**

Correlation between malaria prevalence at antenatal care and among children younger than 5 years was high (r≥0·83 for both surveys), although declines in prevalence at antenatal care were generally smaller than among children. Consistent heterogeneity (p<0·05) in antenatal care prevalence at the district level was evident in all but one region (Kilimanjaro). Data from antenatal care showed declining prevalence in three regions (Arusha, Kilimanjaro, and Manyara) where surveys estimated zero prevalence.

**Interpretation:**

Routine antenatal care-based screening can be used to assess heterogeneity in transmission at finer resolution than population-based surveys, and provides sample sizes powered to detect changes, notably in areas of low transmission where surveys lack power. Declines in prevalence at antenatal care might lag behind those among children, highlighting the value of monitoring burden and continuing prevention efforts among pregnant women as transmission declines. The pregnancy-specific benefits and cost-effectiveness of antenatal care-based screening remain to be assessed.

## Introduction

In 2015, after unprecedented reductions in malaria burden since 2000 that were driven primarily by the large-scale uptake of effective treatment and insecticide-treated nets, WHO set ambitious global targets for further progress towards malaria control and elimination within its Global Technical Strategy for Malaria 2016–30.^1^ However, since 2016, progress towards reducing malaria has stalled, with the estimated global burden rising from 212 million in 2015 to 219 million in 2017.^2^

Surveillance forms a key pillar of the Global Technical Strategy for Malaria 2016–30, with more accurate and timely measurement of malaria burden needed to assess progress, assess the effectiveness of control measures, and more effectively allocate resources.^1^ Many countries have implemented standardised, electronic reporting systems, providing accessible health-facility-level malaria case data rapidly. However, interpretation of these data is challenging owing to differences in reporting and testing practices between settings, and difficulties in determining denominator populations.^3^ Hence, routine surveillance data are judged to be too unreliable by WHO to estimate malaria cases in all but nine countries in mainland Africa (Botswana, Eritrea, Ethiopia, The Gambia, Mauritania, Namibia, Rwanda, Senegal, and Zimbabwe).^2^ Instead, 86% of the global burden estimate of and 90% of malaria deaths are modelled using measures of infection prevalence, most often obtained from population-based household surveys.^4^ The expense of these surveys means that they are only carried out periodically, are generally only powered to estimate prevalence at the level of the first administrative unit, and, because transmission declines to low levels, do not have the power to detect trends. To date, global malaria surveillance programmes do not have adequate tools to monitor malaria exposure among pregnant women, a core risk group owing to the increased risk of maternal and fetal complications— with 54·7 million pregnancies occurring annually in areas of sustained transmission.^5–11^

Quantifying malaria transmission is particularly important for countries with a high burden of malaria, such as Tanzania, which had the fifth highest number of estimated malaria deaths in 2017.^2^ In Tanzania, 93% of the population live, and 1·7 million pregnancies occur annually, in areas where malaria is transmitted,^12^ and transmission is heterogeneous: prevalence in children aged 6–59 months ranged from 0% in highland regions to 25% in western regions in 2017.^13,14^

Since 2007, Tanzania has scaled up testing and treatment for malaria via rapid diagnostic tests and treatment with artemisinin combination therapy (ACT),^15^ distributed 38·9 million long-lasting insecticide-treated nets, and done indoor residual spraying in the Lake Zone.^2^ With these interventions, the national prevalence among children younger than 5 years was halved from 14·4% in 2015–16 to 7·3% in 2017.^13,14^ This decline in transmission is not yet captured within WHO estimates of burden, because the most recent parasite prevalence maps upon which these estimates are based were generated before the publication of the 2017 survey, highlighting the need for more timely measures of prevalence.

In 2014, Tanzania implemented a Single Screen and Treat in pregnancy policy for sentinel population surveillance on the mainland. Under this policy, women are tested at their first antenatal care visit with malaria rapid diagnostic testing, provided with effective treatment (quinine in the first trimester and ACT in the second and third trimesters) if positive, and otherwise receive intermittent preventive therapy in pregnancy per WHO guidelines.^16,17^

Evidence for the utility of antenatal care prevalence as a measure of established metrics of transmission is increasing. In the Democratic Republic of the Congo, Hellewell and colleagues^18^ showed a predictive, although lagged and highly non-linear, relation between clinical incidence of malaria in children and routinely measured antenatal care prevalence. Willilo and colleagues^19^ reported a high degree of correlation in the prevalence of parasitaemia between pregnant women at first antenatal care visit and that among infants presenting for measles vaccination in the Lake Zone, Tanzania. In a review by van Eijk and colleagues,^20^ prevalence of malaria among pregnant women, generally measured as a random community-based sample at a single timepoint, rather than routinely at antenatal care, was highly correlated with that among children younger than 5 years—although prevalence in pregnancy was substantially lower than in children in areas of high transmission.

Given that 98% of pregnant women attend antenatal care at least once in Tanzania, antenatal-care-based testing could be a pragmatic source of sentinel surveillance data to continuously monitor trends in prevalence at much higher spatial and temporal resolution than previously possible, in addition to providing a valuable tool to monitor exposure of pregnant women to malaria infection.

Here, we aimed to describe the results of antenatal-care-based testing in Tanzania during 2014–17. By matching these data by location and timing to two nationally representative prevalence surveys, we provide the first assessment, to our knowledge, of sentinel surveillance within routine antenatal care as a reliable indicator of prevalence trends in children over consecutive surveys.

## Methods

### Data sources

All health facilities in Tanzania (with the exception of high-level facilities, such as hospitals, which upload data directly to District Health Information System 2 [DHIS2]) enter monthly summary reports on antenatal care testing—including the total number of antenatal care attendees, the number of these tested at first visit, and the number testing positive for malaria—into the DHIS2 platform. Data are disaggregated into two age categories (<20 years and ≥20 years); data on gravidity are not entered into DHIS2. District council health management teams do quarterly data quality assessments to monitor and improve data quality and completeness (appendix p 2).^17^

Health facilities, encompassing all locations where heath care is provided, include hospitals, dispensaries, heath centres, and clinics in the public and private sectors.

Ethical clearance was sought and obtained from the Medical Research Coordination Committee of the National Institute for Medical Research.

Data from pregnant women attending their first antenatal care appointment between Jan 1, 2014, and Dec 31, 2017, from all 184 districts of Tanzania (8291 facilities) were obtained from the Health Management Information Systems, which uses the DHIS2 platform. We excluded facilities with no recorded antenatal care attendees during the time period.

Data on malaria prevalence among children aged 6–59 months were obtained from two published, nationally representative, cross-sectional, population-based household surveys: a Demographic and Health Survey done between Aug 22, 2015, and Feb 14, 2016, and a Malaria Indicator Survey done between Oct 9 and Dec 20, 2017. The appendix (p 5) shows the locations of antenatal care clinics and clusters sampled for the 2015–16 Demographic and Health Survey.

### Data processing and analysis

We downloaded facility-level antenatal care data from the DHIS2 system. We assessed malaria prevalence, analysed trends over time, and compared by regions and districts. For trends in prevalence at antenatal care, we report the unadjusted population prevalence of infection by malaria rapid diagnostic testing among women tested at first antenatal care visit (overall and disaggregated by age). We adjusted national and regional-level trends to account for differential uptake of testing across the country by extrapolating monthly prevalence at the district level in those tested to all attendees within the district. In cases where no women were tested within a district in a month (0·2% of district-months), prevalence for standardisation was set to the mean of prevalence from the preceding and subsequent months or the nearest month if either of these also had zero testing (0·05% were based upon prevalence 2 months apart). We obtained contemporary national-level rainfall estimates from the Climate Hazards group Infrared Precipitation with Stations dataset to provide a visual representation of transmission seasons.^21^

To account for seasonal fluctuations in transmission, we compared survey-based prevalence in children younger than 5 years with antenatal care data collected during the period in which the survey was done in each region. As the 2015–16 Demographic and Health Survey was done over a 7-month period, regional antenatal care prevalence was weighted by multiplying by the proportion of Demographic and Health Survey samples collected each month within the region to account for seasonal fluctuations in prevalence. The 2017 Malaria Indicator Survey was done over 3 months, with antenatal care prevalence calculated as the regional mean during those months. Correlation between malaria prevalence among children younger than 5 years in the two surveys (2015–16 and 2017) and that among pregnant women at first antenatal care during the same time periods was then assessed using Pearson’s correlation coefficient (r).

To assess whether malaria exposure during pregnancy varied consistently at the district level over time, we calculated Kendall’s correlation coefficient for monthly district-level malaria prevalence at antenatal care during 2016–17 for each region. This coefficient provides a summary of the extent to which the prevalence ranking of each district remains consistent over time (1 indicates that rankings are consistent throughout the period, 0 reflects an entirely random ranking). We tested the hypothesis that the ordering of district-level prevalence is entirely random within each region. We also report between-survey antenatal care prevalence temporal trends in the three regions (Arusha, Kilimanjaro, and Manyara) in which the estimated prevalence in children in both surveys was zero^13,14^ and the statistical significance of these temporal trends accounted for district-level and seasonal (12-monthly) random effects.

We calculated 95% CIs for non-zero regional prevalence from survey data using a scaled Χ^2^ distribution for the log-likelihood of a binomial distribution implemented through the Survey package in R (version 3.6.0), incorporating the sample design and weighting of each survey. For regions recording zero prevalence, we used unweighted binomially-distributed 95% CIs.

### Role of the funding source

The funder of the study had no role in study design, data collection, data analysis, data interpretation, or writing of the report. The corresponding author had full access to all the data in the study and had final responsibility for the decision to submit for publication.

## Results

We collected data from the Health Management Information Systems and DHIS2 on pregnant women attending their first antenatal care appointment between Jan 1, 2014, and Dec 31, 2017, from all 184 districts of Tanzania (8291 facilities). Of these, 1835 (22%) facilities were excluded for having no recorded antenatal care attendees during the time period (appendix p 3).

**Figure 1:**
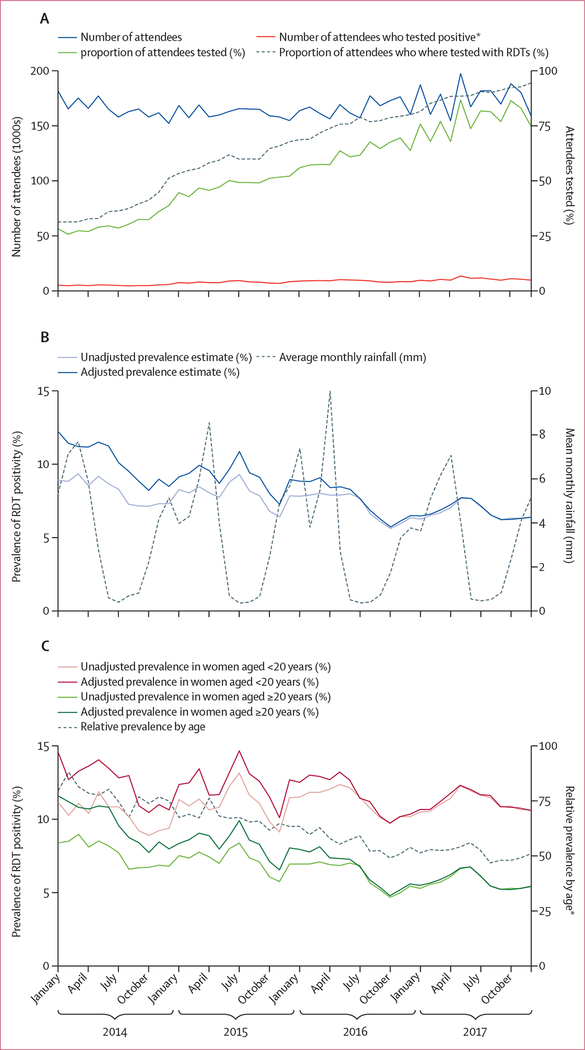
Nationwide expansion of testing and prevalence of malaria at first antenatal care in Tanzania (A) Number of women attending antenatal care, number tested, and number testing positive each month, reported within the Health Management Information Systems. (B) Prevalence of malaria test positivity within women attending antenatal care. Prevalence was adjusted for patterns in uptake of testing at the district level. Prevalence measured by the 2015–16 Demographic Health Survey (14·4%) and 2017 Malaria Indicator Survey (7·3%) are indicated for comparison. Mean monthly rainfall was weighted by the number of women attending antenatal care at the regional level. (C) Prevalence of malaria test positivity in women attending antenatal care, stratified by age. RDT=rapid diagnostic test. *Relative prevalence in women aged ≥20 years as a percentage of that in women aged <20 years.

**Figure 2:**
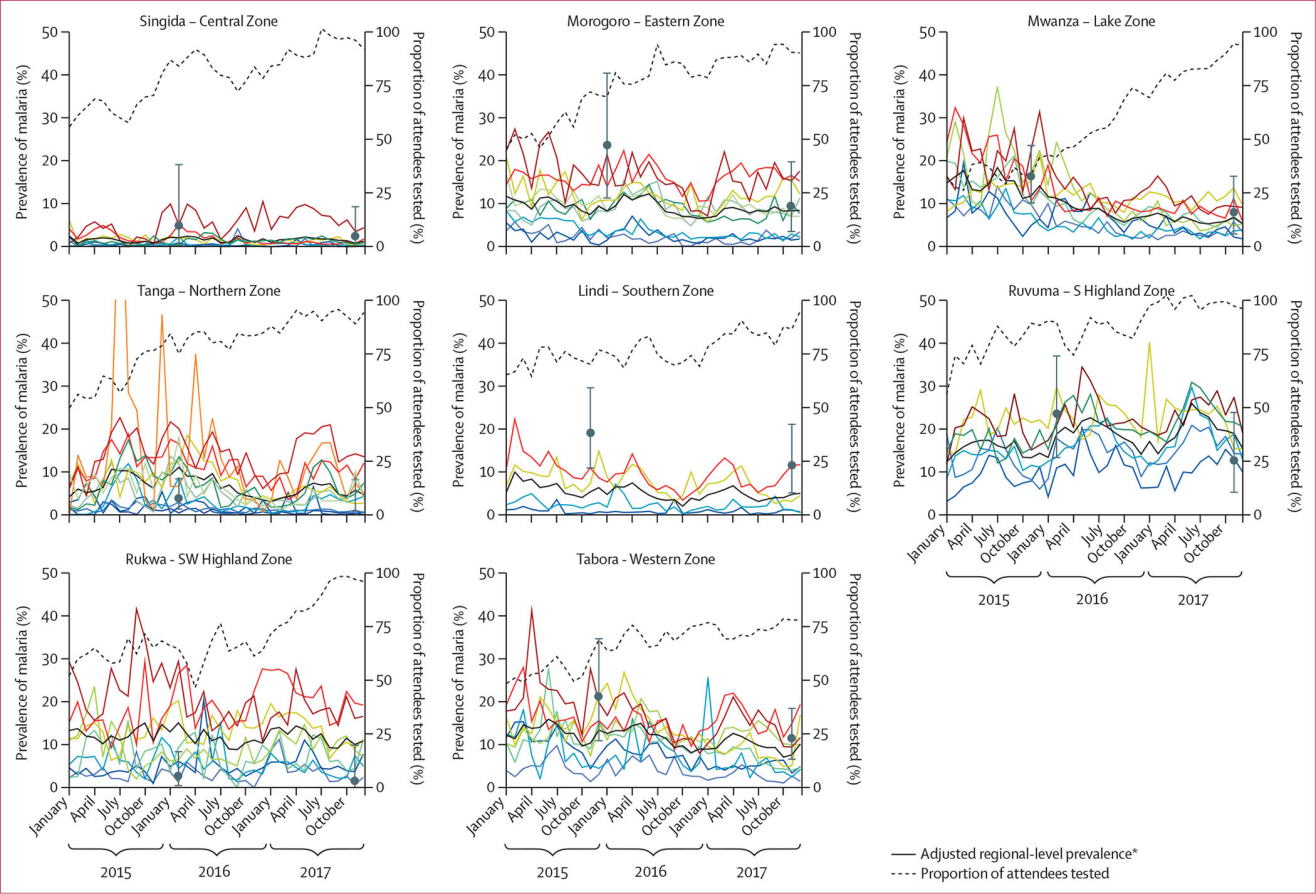
Temporal trends in malaria prevalence at antenatal care by district in selected regions in Tanzania, 2015–17 Coloured lines show mean district-level prevalence and are coloured according to mean prevalence (from dark red representing highest prevalence to dark blue representing lowest). Grey dots and bars show prevalence and 95% CI among children aged <5 years old in the Demographic and Health Survey and Malaria Indicator Survey, with 2015–16 surveys plotted at the month during which the median sample was collected within the region and the Malaria Indicator Survey plotted at November, 2017, the midpoint of the survey. Equivalent figures for all regions grouped by zone are included in the appendix (p 6). *Adjusted for district-level uptake in testing.

In 2014, 71 471 (92% of expected) monthly facility reports were submitted, improving to 77 078 reports (98% of expected) in 2017. Between 2014 and 2017, 7 976 458 pregnant women attended their first antenatal care visit; 20·1% of these were younger than 20 years (table). A median of 164 132 women started antenatal care each month, ranging from 151 172 to 196 181 women per month (figure 1A). Overall, 65·4% of pregnant women were tested for malaria during their first antenatal care visit; this proportion increased from 36·7% in 2014 to 88·6% in 2017. By the end of 2015, over 50% of women were tested in all regions except the Lake Zone, Simiyu, Mwanza, and Geita regions (figure 2, appendix p 6). Testing rates and uptake did not differ by age (appendix p 6).

There was an absolute decline of 1·4% in the overall proportion of women testing positive over the time period, from 8·1% in 2014 to 6·7% in 2017, a proportional reduction of 17% (table). When adjusted to account for the slower uptake of testing in higher-endemicity settings (ie, Lake Zone; figure 2), estimated prevalence among all attendees fell from 10·3% in 2014 to 6·8% in 2017, a proportional reduction of 34·2%. Antenatal care prevalence trends support the hypothesis that the higher prevalence observed in the 2015–16 survey than in the 2017 survey was, to an extent, driven by the attenuated data collection period that extended into the middle of the rainy season, whereas the 2017 survey was done entirely within the dry season. However, adjusted prevalence fell consistently year-on-year, while still displaying typical seasonal patterns throughout the year, suggesting sustained declines in transmission (figure 2, appendix p 7).

There was substantial heterogeneity in prevalence trends by age group (figure 1C). Unadjusted and adjusted prevalence among women younger than 20 years was substantially higher than that among older women and showed smaller reductions over time: adjusted prevalence decreased from 12·6% to 11·2% among younger women, and from 9·7% to 5·7% among older women (table).

**Figure 3:**
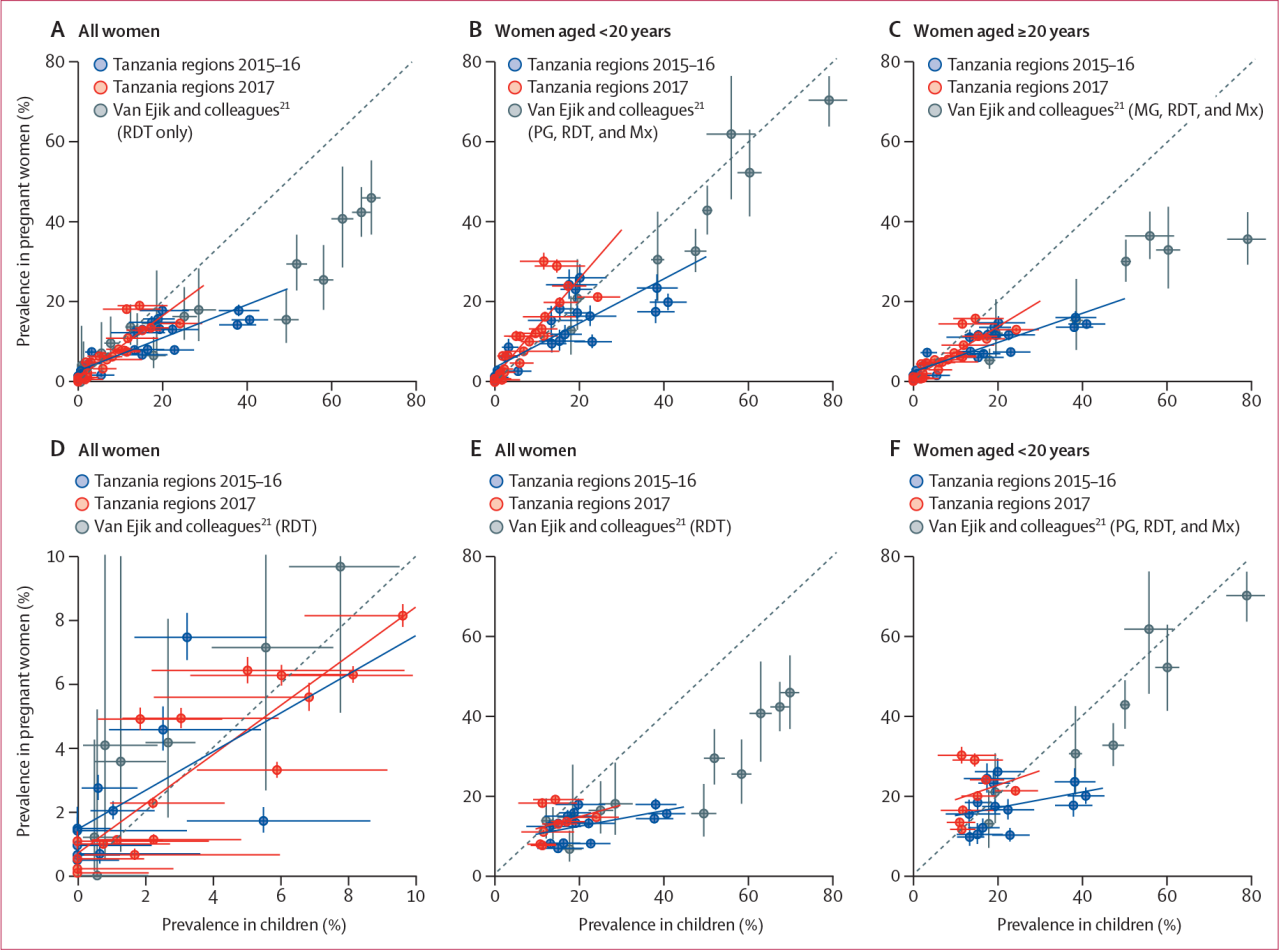
Correlation between population-based surveys and prevalence at antenatal care in Tanzania Lines represent the lines of best fit according to linear least-square regression. Error bars are 95% CIs. (A) Correlation between survey and prevalence at antenatal care among all women (r=0·83, p<0·001). Survey data from 2015–16 (r=0·86, p<0·001) and 2017 (r=0·88, p<0·001) are shown, with correlation by RDT in other surveys from van Eijk and colleagues.^21^ (B) Correlation between survey and prevalence at antenatal care among women aged <20 years (r=0·77, p<0·001), stratified by 2015–16 (r=0·82, p<0·001) and 2017 (r=0·87, p<0·001) surveys, with data from surveys in PG from van Eijk and colleagues. (C) Correlation between survey and prevalence at antenatal care among women aged ≥20 years (r=0·86, p<0·001), stratified by 2015–16 (r=0·88, p<0·001) and 2017 (r=0·89, p<0·001) surveys, with data from surveys in MG from van Eijk and colleagues. (D) and (E) show data from (A) stratified according to whether population-based survey prevalence data are below 10% (D; overall r=0·83, p<0·001) or above 10% (E; overall r=0·37, p=0·09). (D) Data are stratified by 2015–16 (r=0·71, p=0·02) and 2017 (r=0·89, p<0·001) surveys. (E) Data are stratified by 2015–16 (r=0·51, p=0·06) and 2017 (r=0·31, p=0·45) surveys. (F) Comparison in women aged <20 years restricted to regions with survey prevalence >10% (r=0·14, p=0·53), stratified by 2015–16 (r=0·34, p=0·23) and 2017 (r=0·24, p=0·58) surveys. RDT=rapid diagnostic test. Mx=microscopy. PG=primigravidae. MG=multigravidae.

There was good correlation at the regional level between data from children younger than 5 years in population surveys and prevalence at antenatal care; overall (r=0·83), as well as by year (r=0·86 for 2015–16 survey and r=0·88 for 2017; figure 3). Notably, although the degree of correlation was comparable across surveys, the slope of the best-fitting linear least square regression was substantially closer to equality for the 2017 survey than for the 2015–16 survey (figure 3). This finding reflects that regions with higher prevalence (>10%) in 2015–16 declined to a greater extent in children younger than 5 years than in antenatal care attendees between surveys (figure 2). The correlation remains good (r>0·80) for each survey when stratified by age. However, the slope of the best-fitting linear least square regression is substantially higher in younger women than in older women, suggesting that, by the time of the 2017 survey, prevalence in young pregnant women was similar to, or in excess of that in, young children (figure 3). Stratifying survey prevalence by those regions under and above 10% highlights the non-linear nature of the relationship with transmission, with overall antenatal care prevalence providing a close match for under-5 survey prevalence in regions of <10% prevalence (figure 3), but with antenatal care prevalence progressively underestimating under-5 survey prevalence in areas with prevalence >10%, with much lower degrees of correlation (figure 3). Restricting analysis to women younger than 20 years reduced the extent of this underestimate, but did not show the same linear trend seen by van Eijk and colleagues^20^ when restricting analysis to primigravidae (figure 3).

**Figure 4:**
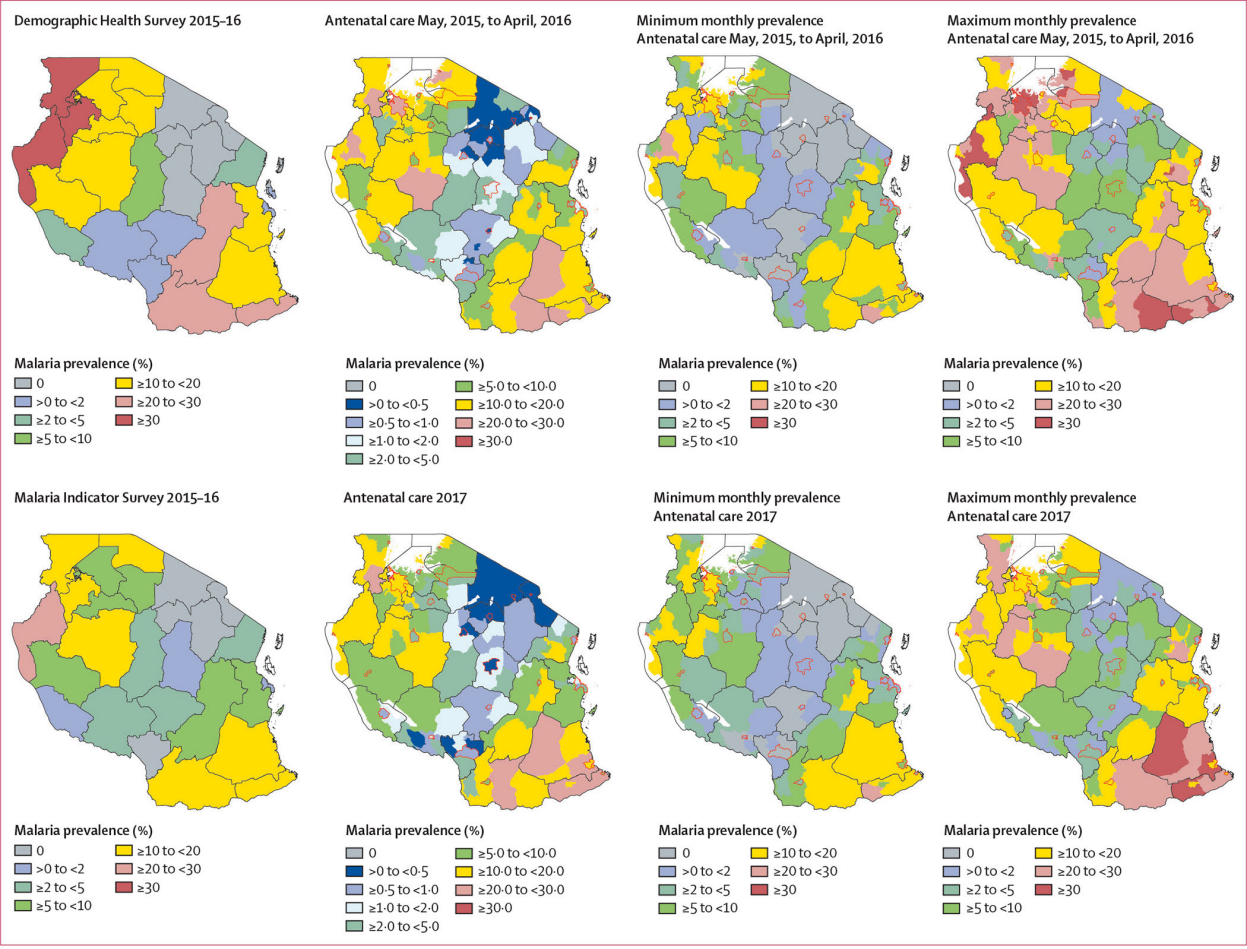
Malaria prevalence by region and method of estimation in Tanzania, 2015–17 Minimum and maximum monthly prevalence refer to the minimum and maximum of the 12 prevalence points recorded on a monthly basis during the indicated year. Increased sample sizes led to increased granularity (ie, more key bands) of some datasets. Region boundaries are shown in black, provincial capitals are shown with red lines; for district-level maps, white areas indicate bodies of water.

Examination of the antenatal care data at the district level highlights substantial and consistent within-region heterogeneity in prevalence among antenatal care attendees (figure 2). All but one region (Kilimanjaro) showed consistent heterogeneity in antenatal care prevalence at the district level (p<0·05; appendix p 4). Mapping these data highlights an association between prevalence and urbanicity, with regional capitals tending to have substantially and consistently lower prevalence than larger, more rural districts (figure 4).

**Figure 5:**
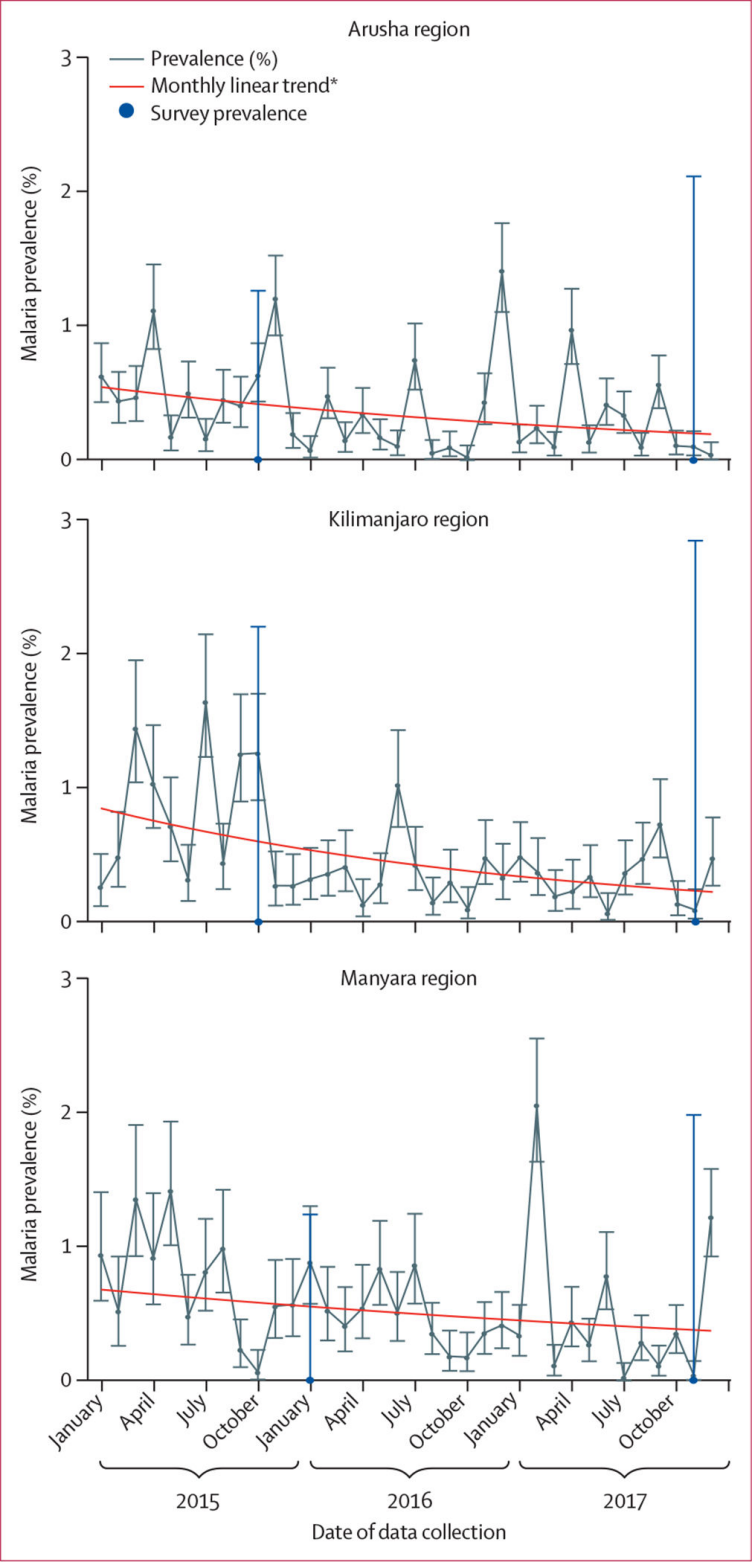
Trends in prevalence at antenatal care in regions with zero prevalence Data are from the Demographic and Health Survey (2015–16) and Malaria Indicator Survey (2017). Error bars are 95% CIs. *Best-fitting monthly linear trend according to logistic regression accounting for random effects at the district level and by month of the year.

Antenatal care prevalence declined in all three regions that recorded zero positive samples in children during both surveys (figure 5). All districts within these regions recorded some level of infection within antenatal care attendees during each of the survey periods.

## Discussion

In this population-based analysis of malaria prevalence in pregnant women in Tanzania, we estimate that prevalence in women routinely tested at antenatal care has fallen steadily since 2014. Our estimate of a decline in antenatal care prevalence from 9·2% to 6·8% between 2015 and 2017 (a 26·1% reduction) contrasts with model-based estimates of burden trends used to assess national progress towards WHO Global Technical Strategy for Malaria 2016–30 targets, which suggest that malaria cases have increased by 0·6% over the same period.^2^ Given that these models are based on data from population-based surveys, future iterations of these estimates might show declines in burden once they have incorporated the 2017 Malaria Indicator Survey, which showed a decrease of about 50% in prevalence since the 2015–16 survey.^13^ Since our estimated declines in prevalence at antenatal care were less substantial than in under-5 survey data, our analysis suggests that further research is required before antenatal care prevalence can be incorporated into such models. However, our analysis highlights the value of this additional, continuous source of prevalence data to provide a more responsive insight into progress and wider transmission trends between surveys and to provide a measure of malaria in pregnancy risk, which does not scale linearly with burden in the general population. Our finding that antenatal care prevalence is more closely aligned to prevalence in children younger than 5 years in areas of low transmission than in areas of high transmission is of particular importance, because these settings are the most difficult and expensive to measure with precision through population-based surveys. Our results also suggest that, in areas approaching elimination, antenatal care prevalence could be a much better indicator than survey data of the presence or absence of asymptomatic infection within regions and districts.

The high degree of within-region heterogeneity in antenatal care prevalence highlights the large efficiencies that are likely when tailoring interventions at a spatial resolution beyond that observable in prevalence surveys. Antenatal care-based surveillance for malaria is not the only putative approach for improving malaria surveillance. Both malaria case counts and test positivity rates among febrile individuals seeking care are increasingly being used to stratify areas of endemicity and to assess ongoing intervention effectiveness.^22,23^ An advantage of these approaches over antenatal care-based surveillance is that they rely upon existing standard malaria case reporting systems. However, interpreting trends in malaria burden from individuals attending health facilities with suspected malaria is challenging, particularly in the context of wider health system strengthening. In mainland Tanzania, total presumed or confirmed cases decreased by 27·7% from 7·7 million cases in 2015 to 5·6 million cases in 2017, but the proportion of confirmed cases has increased from 56·3% to 95·6% during this time period, so that the number of confirmed cases increased from 4·4 to 5·4 million cases.^1^ Use of test positivity rates involves a combination of incidence of febrile malaria and prevalence of asymptomatic malaria infection in attendees with non-malaria-attributable febrile illness, thus is heavily influenced by care-seeking behaviour and the prevalence of other causes of fever.^22^ In Tanzania between 2015 and 2017, although malaria prevalence in children younger than 5 years dropped substantially, overall test positivity rates have risen from 25% to 30%.^2^ By contrast, although routine antenatal care-based surveillance—as a highly accessed, scheduled health system contact and a measure of prevalence—could also be influenced by health system strengthening and reporting rates, it is far less dependent upon health seeking and much more stable with respect to assumptions of denominator populations than measures from individuals with illness accessing care.

Our results also highlight remaining methodological challenges for using antenatal care prevalence as a sentinel surveillance measure of endemicity in the general population. We found that antenatal care prevalence progressively underestimated prevalence among children, as reported previously.^20^ Although trends in prevalence at antenatal care followed seasonal patterns, prevalence at antenatal care in many of the high-transmission settings did not decline to the same extent as that among children between surveys, particularly among pregnant women younger than 20 years. This finding might be attributable to improvements in case management, with younger children more likely to be symptomatic and to receive treatment, and uptake of malaria prevention, because younger women are consistently less likely to use insecticide-treated nets or long-lasting insecticide-treated nets before their first pregnancy.^24^ Age might also be a proxy for gravidity; primigravid women have less immunity to placental-binding phenotypes than do multigravid women, allowing parasites to persist at greater densities in primigravid women and making them easier to detect with rapid diagnostic tests.^6,25^ Genotyping data from Benin showed that malaria infections in pregnancy that were detected by rapid diagnostic testing were often present as submicroscopic infections before conception.^26^ Thus, declines in malaria prevalence might lag behind declines in transmission to a greater extent among pregnant women than among the general population. Future research is required to more accurately quantify the effects of age, gravidity, and gestational age to use antenatal care-based surveillance to accurately estimate prevalence in areas of high transmission, to adjust for other potential sources of bias, such as patterns of long-lasting insecticide treated net uptake and HIV coinfection, and to determine the minimal data required to account for such effects. However, given that pregnant women are a core risk group, being able to track differential dynamics of burden in pregnancy relative to the general population remains a key and unique benefit of collecting these data.

Despite the quality control and assessment measures implemented during the collection period (using the Service Provision Assessment for Malaria tool and the Malaria Services and Data Quality Improvement tool; appendix p 2), the antenatal care-based prevalence data in this study could be affected by various limitations typically associated with routine data, such as imperfect reporting and difficult to quantify errors in data collection, transcription, entry, and collation at all levels. Our prevalence data still provide key insights into malaria dynamics despite these limitations, which are likely to be ubiquitous in all routine data, and highlight the potential generalisability of this form of surveillance—one of the key findings of our study.

There are various limitations associated with the changes in testing uptake throughout the study, particularly in the period immediately after Tanzania’s Single Screen and Treat in pregnancy policy was implemented. In particular, although we have attempted to adjust for the large changes in uptake since 2014, we cannot be certain of the extent to which this adjustment has fully eliminated bias. In particular, if testing in areas of low uptake are prioritised for women with malaria symptoms, this might have artificially inflated our estimates of prevalence at the beginning of the period to an extent that is not possible to adjust for because the data are not disaggregated by clinical symptoms. Conversely, there is currently no means to verify that all women were only tested at their first visit, per policy guidelines, which could also bias later prevalence downwards if this occurred more frequently in the context of higher testing uptake. However, the high correlation with prevalence in children younger than 5 years in 2015–16 and the finding that, subsequently, declines in antenatal care prevalence lagged behind those in children argue against a strong bias in the inter-survey period. The impact of Single Screen and Treat in pregnancy on maternal and fetal outcomes when implemented in conjunction with intermittent preventive therapy in pregnancy has not yet been formally assessed. However, it seems plausible that Single Screen and Treat in pregnancy would provide substantial benefit for women who tested positive, particularly for women presenting to antenatal care in the first trimester before intermittent preventive therapy in pregnancy can be given. In women who present later in pregnancy, the incremental impact is likely to depend heavily on the relative effectiveness of treatment versus the sulfadoxine–pyrimethamine they would otherwise receive. In some parts of Tanzania, the majority of parasites are highly resistant, thus Single Screen and Treat in pregnancy could offer a greater incremental benefit than in areas with less resistant parasites.^24^ Further research is needed to better quantify the added benefit of Single Screen and Treat in pregnancy to the individual woman.

Finally, the benefit of collecting these malaria prevalence data must be weighed against the costs. A previous study^19^ assessing the correlation of malaria prevalence among women attending first antenatal care and children presenting for measles vaccination in Tanzania estimated that about 75% of the direct costs of an Single Screen and Treat in pregnancy policy were owing to purchase and delivery of malaria rapid diagnostic tests. Assuming a cost of US$0·46 per test (SD BIOLINE P.f/Pan; Standard Diagnostic, Suwon City, South Korea)^27^ implies a cost for rapid diagnostic tests of about $1 million annually when given to all pregnant women in Tanzania. This expense has not been restrictive thus far in Tanzania, but must be carefully considered when deciding whether to extend the policy. However, if support for health systems strengthening continues and these data are used wisely to target interventions and track progress, it is likely worth the investment.

## Supplementary Material

Supplementary appendix

## Figures and Tables

**Table:** Annual number of women attending their first antenatal care visits and malaria prevalence, 2014–17

	All visits	Women aged <20 years	Women tested	Prevalence in all women, adjusted (unadjusted)	Prevalence in women aged <20 years, adjusted (unadjusted)	Prevalence in women aged ≥20 years, adjusted (unadjusted)

2014	1 975 237	407 389 (20.6%)	724 256 (36.7%)	10.3% (81%)	12.6% (10.2%)	9.7% (7.6%)
2015	1 930 272	399 554 (20.7%)	1 150 495 (59.6%)	9.2% (8.0%)	12.4% (11.1%)	8.3% (7.2%)
2016	1 978 108	393 507 (19.9%)	1 484 184 (75.0%)	7.6% (71%)	11.7% (11.1%)	6.5% (61%)
2017	2 092 841	402 552 (19.2%)	1 853 938 (88.6%)	6.8% (6.7%)	11.2% (11.1%)	5.7% (5.7%)
Total	7 976 458	1 603 002 (20.1%)	5 212 873 (65.4%)	8.4% (7.3%)	12.0% (11.0%)	7.6% (6.4%)

Data are n or n (% of all visits) unless otherwise specified. Unadjusted estimates of malaria prevalence are the overall proportion of positive tests in women attending antenatal care across the country. Adjusted estimates are those where district-level prevalence in those tested is weighted according to the total number of antenatal care attendees within the district.

## Research in context

### Evidence before this study

We searched PubMed and Google Scholar on July 26, 2019, for literature using the terms “malaria” AND (“pregnancy” OR “antenatal” OR “antenatal care”) AND “surveillance”. We identified three relevant studies, the first study, conducted by van Eijk and colleagues, demonstrated that malaria prevalence data collected from pregnant women attending first antenatal care within research studies and cross-sectional surveys correlate well with data in children younger than 5 years, while the second, by Willilo and colleagues, assessed the correlation between data from pregnant women at first antenatal care with infants 9–12 months of age at measles vaccination in a pilot study in health facilities in one epidemiological zone of Tanzania. The third study from Hellewell and colleagues, which demonstrated that clinical incidence in children younger than 5 years could be used to predict antenatal care prevalence in the context of humanitarian settings, also highlights the potential of antenatal care prevalence as a quantitative metric related to population-level malaria burden.

### Added value of this study

Our study builds upon the work of van Eijk and Willilo and colleagues, to demonstrate that a strong relationship with malaria prevalence in children can also be observed in routine data, collected and processed as part of a nationwide malaria surveillance system. Using data from two nationwide population-based surveys, we are then also able to show that strong correlations exist across multiple timepoints but that declines in antenatal care prevalence in higher transmission regions in Tanzania have lagged somewhat behind those in children. Moreover, monthly prevalence within antenatal care clinics at the district level across all of Tanzania from 2015 to 2018 reveals that substantial, and generally temporally consistent, subregional spatial heterogeneity in prevalence exists beyond the resolution observable in population-based survey data, often corresponding well with known risk factors for malaria transmission such as seasonality in rainfall or urbanicity (visible through the consistently lower levels of transmission within districts representing provincial capitals). Finally, due primarily to the large number of women being tested, we were able to identify spatial and temporal trends in malaria prevalence detected at antenatal visits in regions where population-based surveys consistently do not identify infection—a novel finding that highlights the potential benefits of this form of malaria surveillance in low transmission settings.

### Implications of all the available evidence

There is large potential for routine testing data collected from pregnant women attending first antenatal care to estimate community-level prevalence at much higher spatiotemporal precision than currently available through prevalence surveys, which form the basis of the majority of global malaria burden estimation. Due to the very large numbers of pregnant women, and the high concentration of clinics which provide antenatal care services, including malaria testing throughout the year, antenatal care data could provide estimates of seasonal variation and intensity of transmission in a highly granular fashion, allowing better triangulation between prevalence surveys and routine case reporting systems. However, our finding that declines in prevalence at antenatal care appeared to lag behind those in children in Tanzania suggests that further research is needed before antenatal care prevalence can be used to supplement survey prevalence data in children, which currently largely underpin WHO estimates of malaria burden in Tanzania. Finally, due to the large numbers of women assessed, antenatal care prevalence data appear more sensitive for the detection of prevalence trends in areas of low prevalence than the standard Malaria Indicator Survey and Demographic and Health Survey; thus they might be particularly useful for monitoring in areas approaching elimination.
