# Supplementary material for: Estimating malaria burden among pregnant women using data from antenatal care centres in Tanzania: a population-based study
Source: Lancet Glob Health. Author manuscript; Available in PMC 2022 Jan 11. (PMC8752309; doi:10.1016/S2214-109X(19)30405-X)
Supplement: Supplementary appendix [file NIHMS1764586-supplement-Supplementary_appendix.pdf]

THE LANCET
Global Health

Supplementary appendix

This appendix formed part of the original submission and has been peer reviewed. We post it as supplied by the authors.

Supplement to: Kitojo C, Gutman JR, Chacky F, et al. Estimating malaria burden among pregnant women using data from antenatal care centres in Tanzania: a population-based study. *Lancet Glob Health* 2019; **7:** e1695–705.

Supplementary appendix

Kitojo C, Gutman JR, Chacky F, et al.: Estimating malaria burden among pregnant women using data from antenatal care centres collected between 2014 and 2017 and implications for elimination strategies in Tanzania: a population-based study

Table of Contents

Supplemental Methods...2
S1.1 DHIS2 data flow and data Quality 2
Table S1. Routine Indicators Captured at Antenatal Care 2
S.1.2 Heath facilities with missing ANC data 3
Supplementary References...3
Table S2. Degree of sub-regional spatial structure in prevalence at ANC 2015- 2017..........................4
Supplementary figure: S1. Locations of data collection 5
Supplementary Figure S2: Temporal trends in nationwide testing uptake between 2014 and 2017 stratified by age category 6
Supplementary Figure S3: Temporal trends in prevalence at ANC by region in Tanzania 2015-2017 .. 7

Supplemental Methods

S1.1 DHIS2 data flow and data Quality

According to the National diagnostics and treatment guidelines,[1] all pregnant women attending first ANC should be tested for malaria regardless of symptoms. Those who test positive receive appropriate treatment according to the national guidelines, among other services. Test outcomes are recorded in the ANC register and reported to the respective district on a monthly basis. In the councils, the reports are entered into the dedicated DHIS2 database, and the data are analyzed in the malaria dashboard according to the organizational level. The indicators captured at ANC and their descriptions are shown in Table S1.

Table S1. Routine Indicators Captured at Antenatal Care

**Indicator**	**Description**
Malaria test	Pregnant women attending ANCs for the first time who are tested for malaria (mRDT OR BS)
Positive malaria test	Pregnant women who test positive for malaria malaria by mRDT or microscopy
First ANC attendance<br>- Under 12 weeks<br>- Above 12 weeks	Number of pregnant women attending ANCs for the first time, stratified by gestational age at the time of the initial visit
Test rate	Proportion of pregnant women tested for malaria at ANCs (i.e., Malaria Test/ (First ANC Attendance Under 12 weeks + First ANC Attendance Above 12 weeks)
Positivity rate	Proportion of pregnant women tested for malaria at ANCs who are positive for malaria parasites (i.e., Positive malaria test / Malaria Test)

According to the National Guidelines for Malaria Surveillance and Response from July 2017,[2] ANC data is recorded at health facilities (HFs) and reported monthly to the district in a paper based format. HFs enter data into registers, such as the ANC pregnant women register, tally sheets, and ANC monthly summary reports. At the district level, dedicated health management information system (HMIS) staff enters the data into a web-database, the District Health Information System (DHIS2). Data are ideally analyzed at all health care delivery system levels by automatically generated specific outputs from DHIS2. The data available on DHIS2 can be viewed on the dashboard with different reports formats. Maintaining high quality and timely data is important to ensure the correct data is availed to all levels from the HF to the district and national level. NMCP has developed a data quality system that aims at improving the quality of malaria data in terms of consistency and completeness, and helping district councils and regions to track performance and make decisions on allocation of resources. Quality assurance[3] and quality control (QC) of routine data is conducted routinely using the Malaria Services and Data Quality Improvement (MSDQI) tool developed and rolled out nationally in 2017. Prior to 2017, NMCP conducted

data quality assessments using the Service Provision Assessment for malaria (SPAM) which has the same goals as MSDQI. The MSDQI tool consist of seven modules; ANC, out-patient department (OPD), in-patient department (IPD), data quality assessments (DQA), logistic and supply chain, microscopy and malaria rapid diagnostic tests (mRDTs). The tool is geared to improve the effectiveness of supportive supervision in order to strengthen clinical and diagnostics services by identifying needed improvements in knowledge and skills, and supporting clinical, data, and laboratory staff to address these needs. This involves assessments of availability and adherence to the diagnostic algorithm. It also provides an opportunity for feedback to sub-levels, follow-up and mentorships.

The data quality assessment (DQA) is conducted quarterly and it involves identification of errors, inconsistencies, and other data abnormalities and conducting activities to improve the quality of data, such as on-the-job training and coaching. The DQA identifies issues such as incomplete or inconsistent registers and tally sheets, and immediate actions are taken to improve future performant through coaching and mentorships. Facilities develop clear action plans with clear roles and responsibilities and time frames to improve data management.

S.1.2 Heath facilities with missing ANC data

This study used data from DHIS2 which includes reports from all health facilities from mainland Tanzania, both public and private facilities. A record for each month is generated in the system from each of the 8,219 health facilities, even if the health facility does not offer the service; in this case ANC. We identified 1,835 facilities (22% of the total) with no reported data for any of the ANC indicators for any of the included months; these facilities were excluded. The vast majority of these facilities do not offer ANC at all; at least 63 (3.4%) were dental clinics and at least 33 were eye clinics (1.8%). We reviewed the Ministry of Health Database for 100 randomly selected facilities from the remaining 1,739 facilities with missing data, of those, 95% do not offer ANC services.

Supplementary References

1. Ministry Of Health Community Development, Gender, Elderly, and Children: National Guidelines for Diagnosis and Treatment of Malaria. 2014.
2. Ministry Of Health ,Community Development,Gender, Elderly, and Children:National Guidelines for Malaria Surveillance and Response. 2017.
3. Blencowe H, Chou VB, Lawn JE, Bhutta ZA. Modelling stillbirth mortality reduction with the lives saved tool. BMC public health. 2017;17(4):784.
4. Ministry Of Health Community Development Gender, Elderly, and Children (MoHCDGEC), Tanzania mainland, Ministry of Health (MoH) Zanzibar, National Bureau of Statistics (NBS), Statistician (OCGS), Macro aI. Tanzania Demographic and Heath Survey and Malaria Indicator Survey 2015-16. Rockville, Maryland, USA: MoHCDGEC, MoH, NBS, OCGS, and ICF. 2016.

Table S2. Degree of sub-regional spatial structure in prevalence at ANC 2015- 2017

Zone	Region	No of Districts	Kendall W coefficient**	p-value
**Central**	Dodoma	8	0.513529	<0.0001
**Central**	Manyara	7	0.336716	<0.0001
**Central**	Singida*	7	0.699012	<0.0001
**Eastern**	Dar Es Salaam	5	0.618403	<0.0001
**Eastern**	Morogoro*	9	0.81522	<0.0001
**Eastern**	Pwani	9	0.695313	<0.0001
**Lakes**	Geita	6	0.66369	<0.0001
**Lakes**	Kagera	8	0.557953	<0.0001
**Lakes**	Mara	9	0.517419	<0.0001
**Lakes**	Mwanza*	8	0.75339	<0.0001
**Lakes**	Shinyanga	6	0.477976	<0.0001
**Lakes**	Simiyu	6	0.588294	<0.0001
**Northern**	Arusha	7	0.101516	0.023
**Northern**	Kilimanjaro	7	0.059347	0.201
**Northern**	Tanga*	11	0.744918	<0.0001
**South West Highlands**	Katavi	5	0.335069	<0.0001
**South West Highlands**	Mbeya[¥]	12	0.732316	<0.0001
**South West Highlands**	Rukwa*	4	0.804167	<0.0001
**Southern**	Lindi*	6	0.691468	<0.0001
**Southern**	Mtwara	9	0.671933	<0.0001
**Southern Highlands**	Iringa	5	0.371695	<0.0001
**Southern Highlands**	Njombe	6	0.37341	<0.0001
**Southern Highlands**	Ruvuma*	8	0.696181	<0.0001
**Western**	Kigoma	8	0.493717	<0.0001
**Western**	Tabora*	8	0.58416	<0.0001

*Indicates Region within each zone with most consistent district-level structure which are then plotted in Figure 3 in the main manuscript; all other regions are plotted in Supplement figure S3.
[¥]Combines data for Mbeya and Songwe region.
** Kendall's W coefficient, or Kendall's coefficient of concordance is a non-parametric statistic which assesses the degree to which two measures arrive at the same ranking / ordering of a number of items.

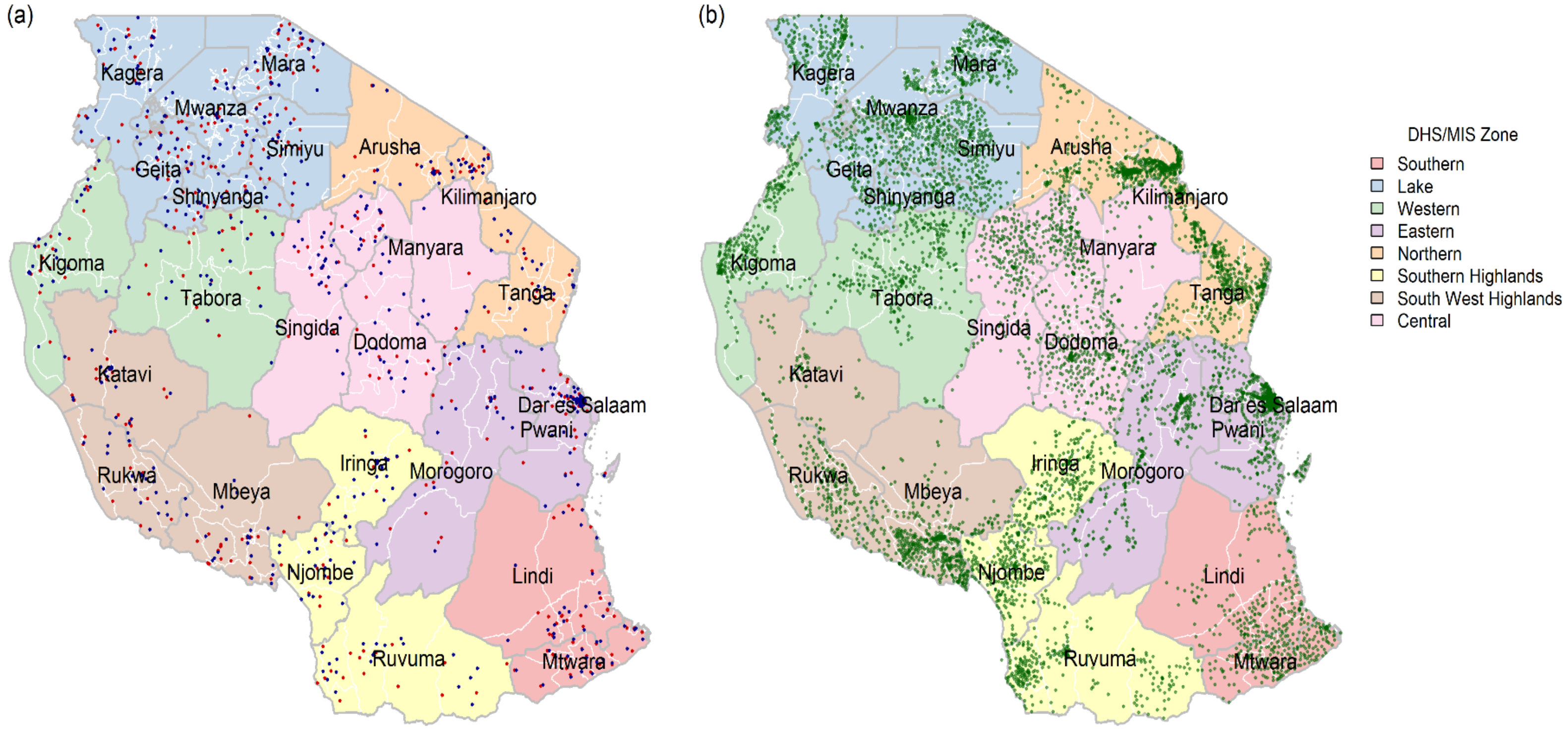

Supplementary figure: S1. Locations of data collection

Geo-locations of clusters sampled during the 2015/2016 DHS (red dots) and 2017 MIS (blue dots) surveys(mapped geo-locations are randomly displaced up to 2 kilometers for urban clusters and 5 kilometers for rural clusters according DHS protocol), (b) geo-locations of ANC facilities routinely reporting monthly testing data. Maps are colour-coded by epidemiological zone as defined within the DHS 2015/16 final report,[4] grey lines show regional boundaries, white lines show district boundaries within regions.

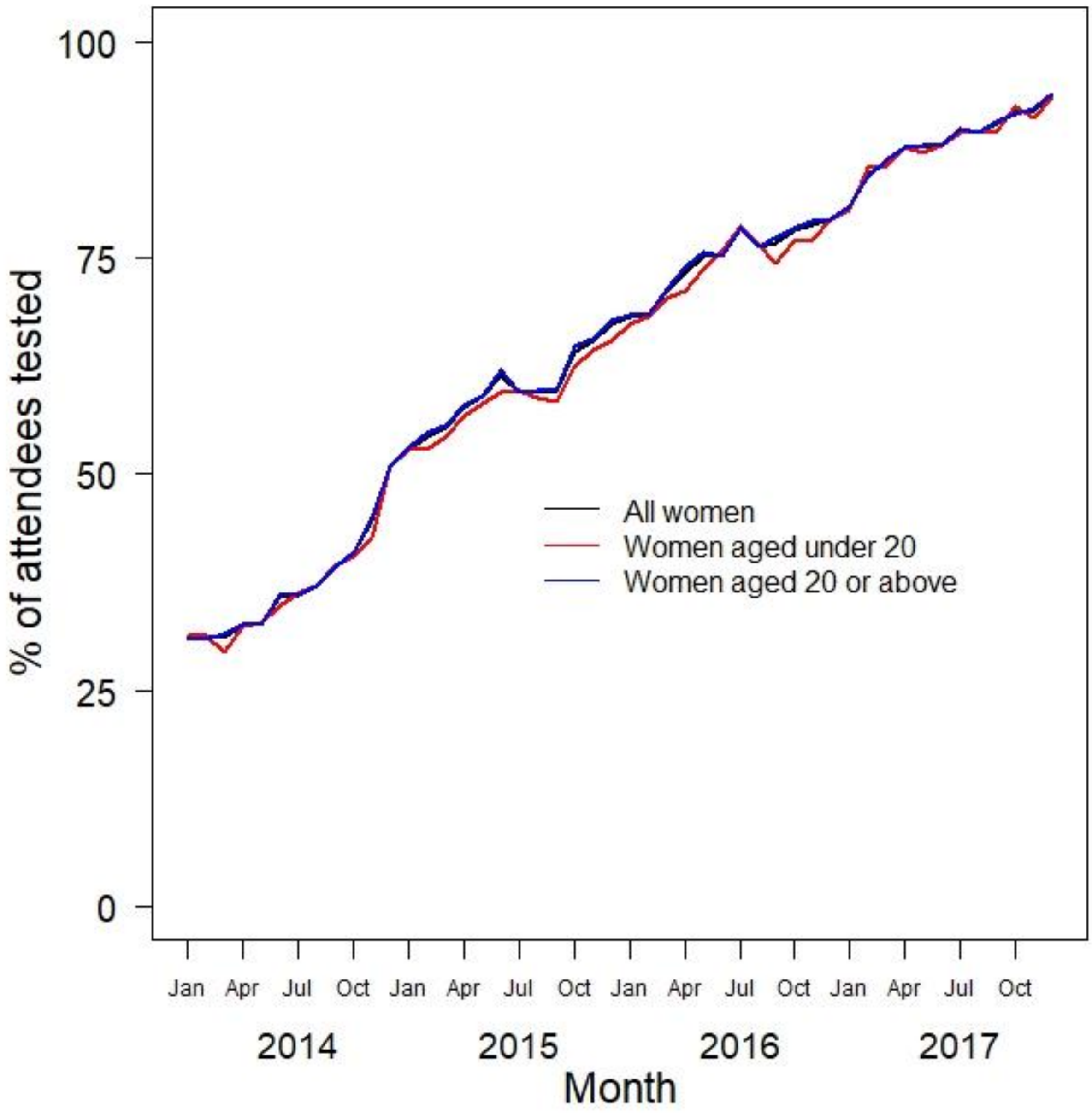

Supplementary Figure S2: Temporal trends in nationwide testing uptake between 2014 and 2017 stratified by age category

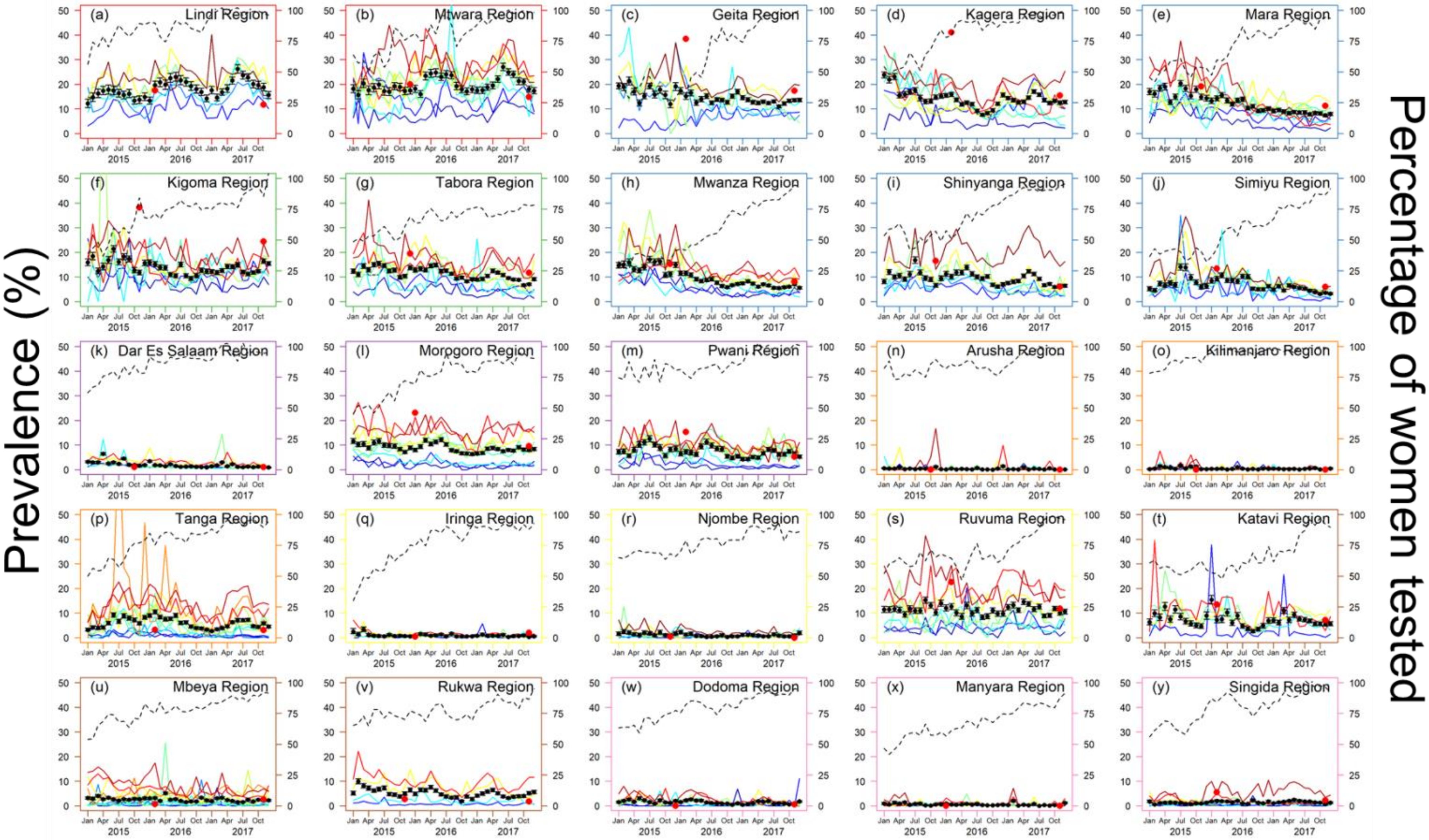

Supplementary Figure S3: Temporal trends in prevalence at ANC by region in Tanzania 2015-2017

Black dots and bars show monthly prevalence and associated 95% confidence intervals. Lines show district-level prevalence and are coloured according to average prevalence (from dark red representing highest prevalence to dark blue representing lowest). Red dots show DHS/MIS prevalence, with 2015/16 surveys plotted at the month during which the median sample was collected within the region. The percentage of total women attending first ANC in each Region who were tested is shown with a dashed black line and plotted according to the right-hand axis. Figures are grouped and outlines colour-coded to reflect the zones of Tanzania (red = Southern, blue= Lakes, green=Western, purple = Eastern, orange = Northern, yellow=Southern Highlands, brown= South West Highlands, pink= Central).
